# A new technology for pacifier weaning: a thematic analysis

**DOI:** 10.3389/fped.2023.1161886

**Published:** 2023-05-24

**Authors:** Ahmed Al Hariri

**Affiliations:** Department of Psychology, College of Arts, Taif University, Taif, Saudi Arabia

**Keywords:** patent, new technology, pacifier weaning, thematic analysis, Saudi Arabia

## Abstract

**Introduction:**

Babies and toddlers often become accustomed to using baby pacifiers. However, pacifiers may harm children's health and lead to various problems, such as less frequent breastfeeding, shorter breastfeeding duration, dental deformities, tooth decay, recurrent acute otitis media, sleep disorders, and the potential for accidents. This study aims to introduce new technology that may prevent babies from becoming used to a pacifier (patent titled “Prevents Getting Used to Pacifier Baby, Number SA10609, Saudi Authority for Intellectual Property”). This study used a descriptive qualitative design.

**Methods:**

The participants included three pediatricians, three psychologists, three dentists, three family doctors, and three mothers of babies and toddlers, with a mean age of 42.6 years old (SD = 9.51). Semi-structured interviews were used, and thematic analysis was conducted to generate a thematic tree.

**Results and Discussion:**

The thematic analysis resulted in three themes: (1) the disadvantages of pacifier use, (2) the introduction of new technology for the patent, and (3) the expectations for this technology. The results showed that a pacifier might negatively affect the health of babies and toddlers. However, the new technology may prevent children from becoming used to pacifiers and protect them from any possible physical or mental issues.

## 1. Introduction

Pacifiers, also known as soothers, dummies, or artificial teats (1), facilitate nonnutritive sucking, which developmental experts widely acknowledge as providing self-comfort, a sense of calm, and control when a baby or toddler feels upset or stressed (1, 2). Some studies have shown the practical benefits of pacifier use, such as a decrease in the possibility of sudden infant death syndrome (SIDS) (2, 3) and adjunctive pain relief (4). These benefits may explain why pacifier use is widespread worldwide (5). Nevertheless, pacifier use is debatable. Some people believe in its benefits, while others stress its drawbacks (6). Several health issues related to pacifier use include breastfeeding problems, ear infections, tooth deformities, speech errors, bacteria-related illnesses, and sleep disorders. The following paragraphs discuss these issues in more detail.

The United Nations Children's Fund and World Health Organization (WHO) are against pacifier use because it leads to early weaning from breastfeeding. The WHO recommends “[giving] no artificial teats or pacifiers to breastfeeding infants” (7). Many healthcare professionals have followed the WHO's instructions (8). Pacifiers use negatively affects breastfeeding and is associated with a reduced duration or cessation of breastfeeding (9–11). Pacifier use also leads to nipple confusion or preference, as babies may prefer pacifier nipples over their mothers’ nipples (8).

There are other issues related to pacifier use. For example, pacifier use is linked to a 1.8-fold increased risk of recurrent acute otitis media in children younger than four. Therefore, parents should be informed about this adverse effect to avoid recurrent episodes (12). In addition, Warren et al. (13) showed significant differences in the dental arches of young children at 24 months and 36 months compared with their peers who stopped sucking before they reached 12 months. Two other studies (14, 15) have confirmed that pacifier use may cause tooth displacement. Evidence indicates that four to six hours per day of pacifier use cause tooth movement, which varies according to the intensity, duration, and frequency of pacifier use (16). It may also increase the number of atypical speech errors in young children (17). Barca (18) suggested that pacifier use should be minimized after the first year of life, especially during the day, because it may affect the processing of proprioceptive information, speech-motor programs, and auditory input. Similarly, Bueno et al. (19) found a significant association between increased atypical errors and the frequency of pacifier use during the daytime.

Pacifier use poses other problems. Pacifiers are constantly colonized with microorganisms and support major biofilms, which can be potential reservoirs of infections (20–22) and cause illnesses. For example, a link between pacifier use and COVID-19 was recently identified (23). The pacifier-antiseptic combination, which is often used to disinfect pacifiers, has been found to lead to a higher risk of subsequent food allergies (24). Sleeping disorders are also associated with pacifier use (25). In a cross-sectional study, Balaban et al. (26) concluded that children who used pacifiers did not have better sleep quality. Similarly, Hanzer et al. (27) found that 40.4% of children who participated in their study (*n* = 36) were awakened when their pacifiers fell from their mouths.

As shown by this discussion, pacifier use has some benefits, but it also poses many risks that may affect the health of babies and toddlers. Some inventors have created devices or methods for pacifier-weaning purposes. For instance, Bashir’s (28) patent titled “Improvements in Baby and Infant Pacifier Weaning Methods, Patent Number WO2016174381A1” relates to a pacifier that uses a discouraging image or miniature model, such as an insect, in a teat or nipple. When children see this pacifier, they may refuse it. Arguably, this invention has two disadvantages. First, children may develop entomophobia [a specific phobia characterized by an excessive or unrealistic fear of one or more insects (29)]. Second, not every child knows the shape of an insect, so a child may still use this pacifier.

Similarly, Parker and Ramundo (30) invented the “Pacifier Weaning Method and Device, Patent Number US8298263B2”, which they described as a device, system, and method that helps a baby gradually stop pacifier sucking. The system includes six pacifiers with different nub sizes. A mother gives her child the size she thinks is suitable. Eventually, the child is given a pacifier with a shorter nub than the previous one. This method is repeated using a smaller size each time until the child is weaned off the pacifier. However, small pacifiers may be swallowed, and although the child is moving to smaller pacifiers, they still spend time using a pacifier, so the sucking habit continues.

With both patents (WO2016174381A1 and US8298263B2), the child may or may not reject the pacifier. The argument is not against these inventions, as they attempt to help with pacifier weaning, but not every child will react to these inventions similarly. Other patents might be better and can be considered as additional attempts to help with pacifier weaning, like the one discussed in the following section

**Figure 1:**
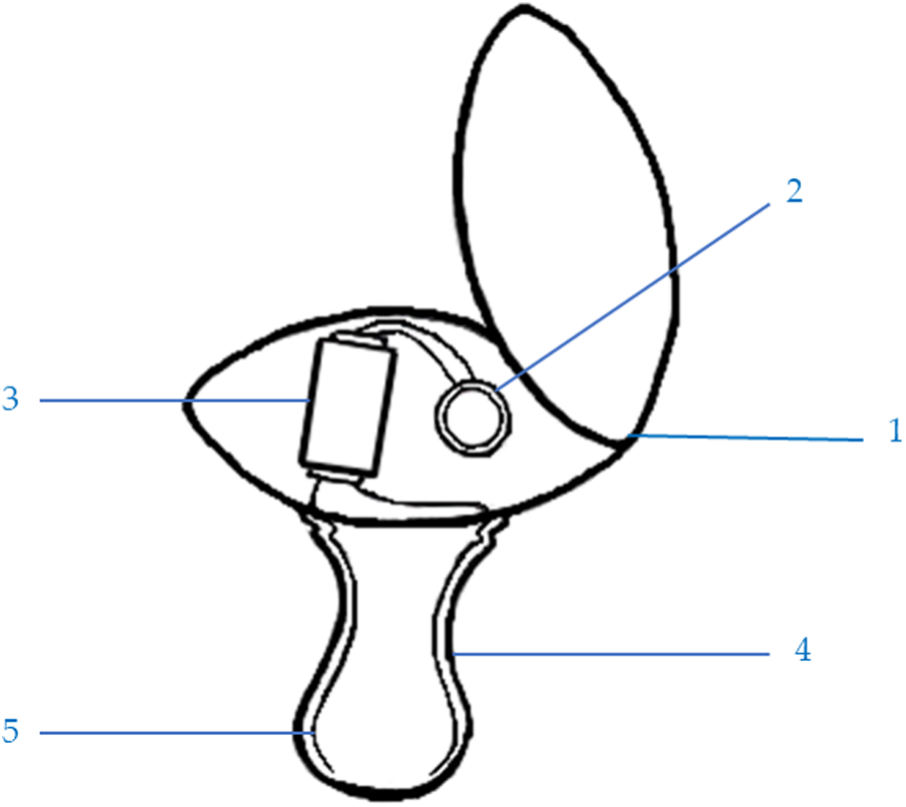
The parts of the patent: (**1**) waterproof base and cover, (**2**) two-volt lithium battery, (**3**) electric shocker, (**4**) pacifier nipple, and (**5**) electric connections.

Al Hariri (31) invented a new technology entitled “Prevents Getting Used to Pacifier Baby, Patent Number SA10609” from the Saudi Authority for Intellectual Property. This patent aims to help stop the habit of pacifier use (i.e., pacifier weaning). It involves an electric shock, which is already used for medical reasons (32, 33), to make the child immediately reject the pacifier because it is improbable that a child will like to be shocked. As shown in Figure 1, the patent includes the following parts:

(1) The round base of the pacifier and its cover, which is waterproof, has a diameter of 3.5 cm and a width of 1 cm.
(2) A battery is in the round base. According to the safety regulations for pacifier use, either a small, round two-volt lithium battery or a four-volt lithium battery can be used.
(3) An electric shocker is located near the battery inside the round base.
(4) A pacifier nipple made from safe rubber or plastic is used.
(5) Electric wires connect the electric poles on the battery and the pacifier nipple with the electric shocker.

**Figure 2:**
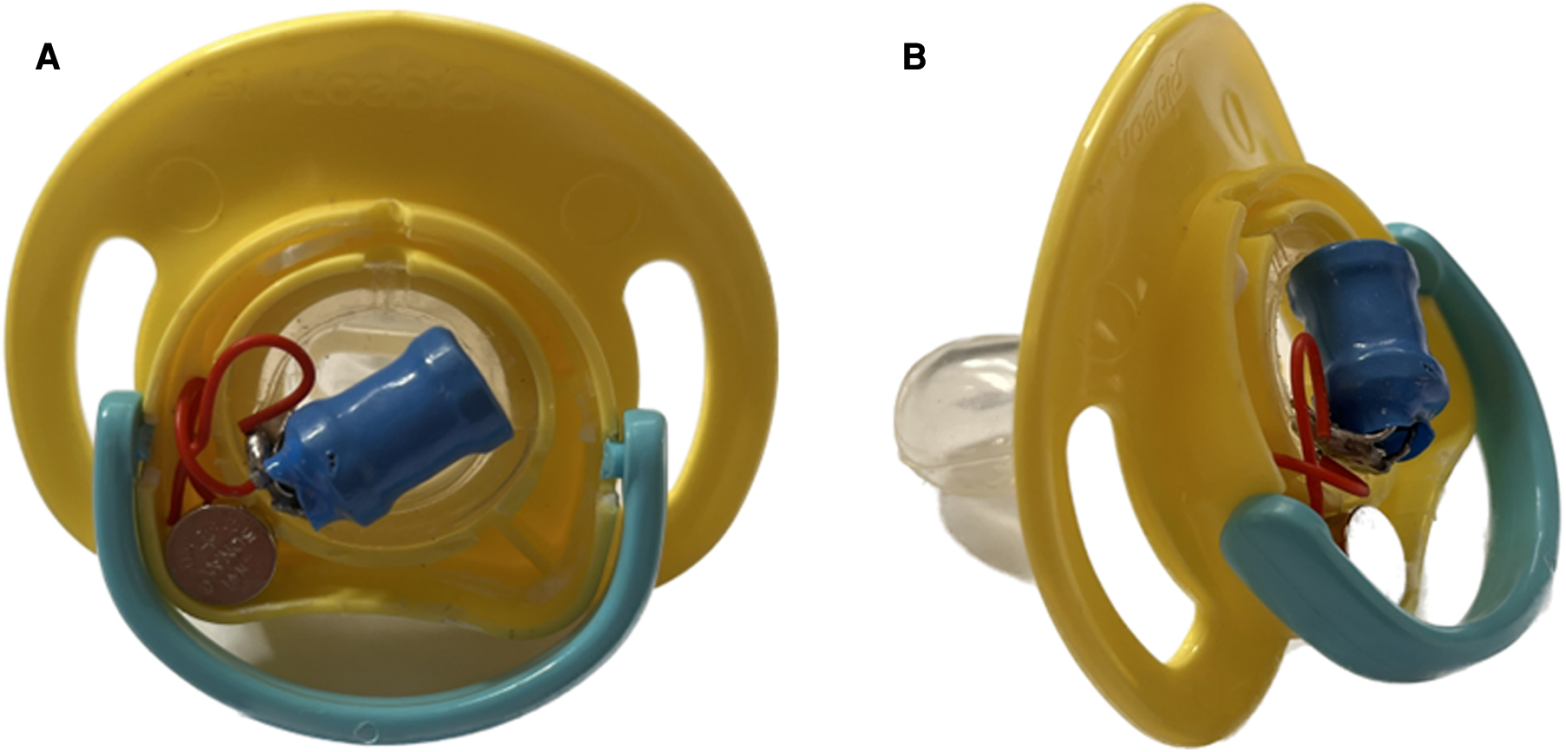
A model of the patents shows (**A**) the patent's technology in the back of the pacifier and (**B**) the patent from the side.

The pacifier operates by using small wires to link the electric shocker (3) to the electric poles (positive and negative) of the lithium battery (2) and the pacifier nipple (4). The exposed wires (5) are on the nipple's wall, so when the pacifier nipple gets wet with baby saliva, it generates an electric shock to the baby's mouth. The lithium battery needs to be changed occasionally by opening the round base cover (1) and replacing the battery. The electric shocker is also replaceable if it breaks. This pacifier can be designed in a lovely, attractive shape and many sizes. Figure 2 presents a model of the patent.

Oral habits like non-nutritive sucking differ in duration and intensity (34). As a result, there is no set timeline for weaning a child off a pacifier, as every child is different (5). Therefore, the duration of the use of the current patent is different from child to child. The patent can be used as much as needed so the child can be weaned. However, it is expected that when a child uses this patent five times and each time last for just a few seconds (without having another regular pacifier around), the child will be weaned from the pacifier. Considering that the shock's intensity will not differ across children from different age groups in the lactation stage, the shock's power ranges between two and four volts based on the battery used. Considering also that using 2–4 volts is ethically acceptable in children (as discussed in Section 4).

To the best of our knowledge, research discussing patents or technologies related to pacifier weaning is scarce. The current study aims to enhance our understanding of the new technology of “Prevents Getting Used to Pacifier Baby, Patent Number SA10609”. The study also sheds light on the disadvantages of pacifier use, introduces the patent, and discusses its benefits and expectations by collecting data from mothers and professionals who treat babies or toddlers who use pacifiers.

## 2. Materials and methods

### 2.1. The study design and study sample

This study adopted a qualitative descriptive design to explore the participants’ perceptions of and experiences with pacifier use and their expectations about the new technology that helps in pacifier weaning. A purposeful sampling method was used to recruit the participants. Specifically, the sample included mothers and health practitioners (psychologists, pediatrics, family doctors, and dentists) who treat babies and toddlers who have become accustomed to using pacifiers. The researcher is a counselor in mental health and recruited the health participants from the clinic where he works. He also recruited mothers who were already clients in the clinic.

### 2.2. Data collection

Semi-structured interviews were used to explore the participants’ experiences with babies who become used to pacifiers and to consider their views regarding the new pacifier-weaning technology. The author developed the interview questions about the new patent using (1) two questions from Joyner et al. (35) interviews that focused on the interviewees’ beliefs about pacifier use and (2) the author's experiences as a mental health counselor, inventor, and researcher. Table 1 shows the interview questions.

**Table 1:** Semi-structured interview questions.

The subject	The questions
The interviewees believe in pacifier use [from Joyner et al. (35)].	• How do you feel about pacifiers?
	• What are the advantages or disadvantages of using a pacifier?
The interviewees believe in the new patent.	• What do you think about this new patent?
	• Do you think this patent is valuable and helpful in child pacifier weaning?
	• What do you expect of this new patent?

The interviews were conducted as regular discussions to help the interviewees relax and speak freely. This ease of dialogue helps reduce subjectivity or bias in the collected qualitative data (36). In addition, it was found that if the respondents are familiar with the interviewer (as is the case with the current participants), they will feel more comfortable answering the interview questions (37–39), which makes the current data valid and trustable. Each interview began with questions about the participant's experience with a child (or children) who uses a pacifier and the participant's perception of whether a pacifier is valuable and why. After that, the patent was introduced and explained to the participant by showing Figures 1, 2. The participant's notions and expectations about this new technology were then discussed. The interviews were conducted in the clinic where the author works. Each interview lasted approximately 15–20 min and was audio recorded.

### 2.3. Data analysis

The audio recordings of the interviews were transcribed literally and then translated from Arabic (the participants’ mother tongue) into English. After that, the data were imported into ATLAS.ti (40), a qualitative software program used for thematic content analysis. Thematic analysis was adopted for data analysis because it identifies patterns and captures the complexities of meaning within qualitative data (41, 42). As suggested by Braun and Clarke (43), the following steps were followed:

(1) The researcher read and re-read all transcripts to become familiar with the data.
(2) Relevant data were grouped into similar codes.
(3) The codes were categorized into possible pacifier-related topics.
(4) The codes and data set were confirmed and checked to ensure thematic validity.
(5) Topics were defined in a final thematic tree. To ensure data consistency, the author took notes about the interviewee’s body language, reactions, and the main points they stressed after each interview.

### 2.4. Ethics

This study was conducted after receiving approval from the Scientific Research Ethics Committee of Taif University (protocol code HAO-02-T-105 and date of approval 30/10/2022). All participants were informed about the study's purpose and provided written consent. To ensure confidentiality, all sociodemographic data, interview records, and transcriptions were saved and password-protected, accessible only by the author. None of the participants’ real names were used in this study. Instead, all the presented interview excerpts use phrases such as “Pediatrician 1” or “Dentist 2”.

## 3. Results

### 3.1. Sample description

Fifteen people participated in this study. The sample included seven males and five females and included pediatricians, psychologists, dentists, family doctors, and mothers. As presented in Table 2, the participants’ ages varied from 22 to 61 (*M*_age_ = 42.6; SD_age_ = 9.51).

**Table 2:** Participants’ demographics.

Age	Gender	Position/Participant label*
46	M	Pediatrician 1
51	M	Pediatrician 2
38	F	Pediatrician 3
39	F	Psychologist 1
56	M	Psychologist 2
44	M	Psychologist 3
47	M	Dentist 1
45	F	Dentist 2
39	F	Dentist 3
43	M	Family doctor 1
39	M	Family doctor 2
61	F	Family doctor 3
38	F	Mother 1
31	F	Mother 2
22	F	Mother 3

* Participant labels are used for the interview extracts in Sections 3.2.1–3.2.3.

All the participants interact with babies or toddlers who use pacifiers. The three mothers are responsible for children aged five months to two years old who use pacifiers. Health practitioners see babies and toddlers who use pacifiers in their clinics.

### 3.2. Qualitative findings

**Figure 3:**
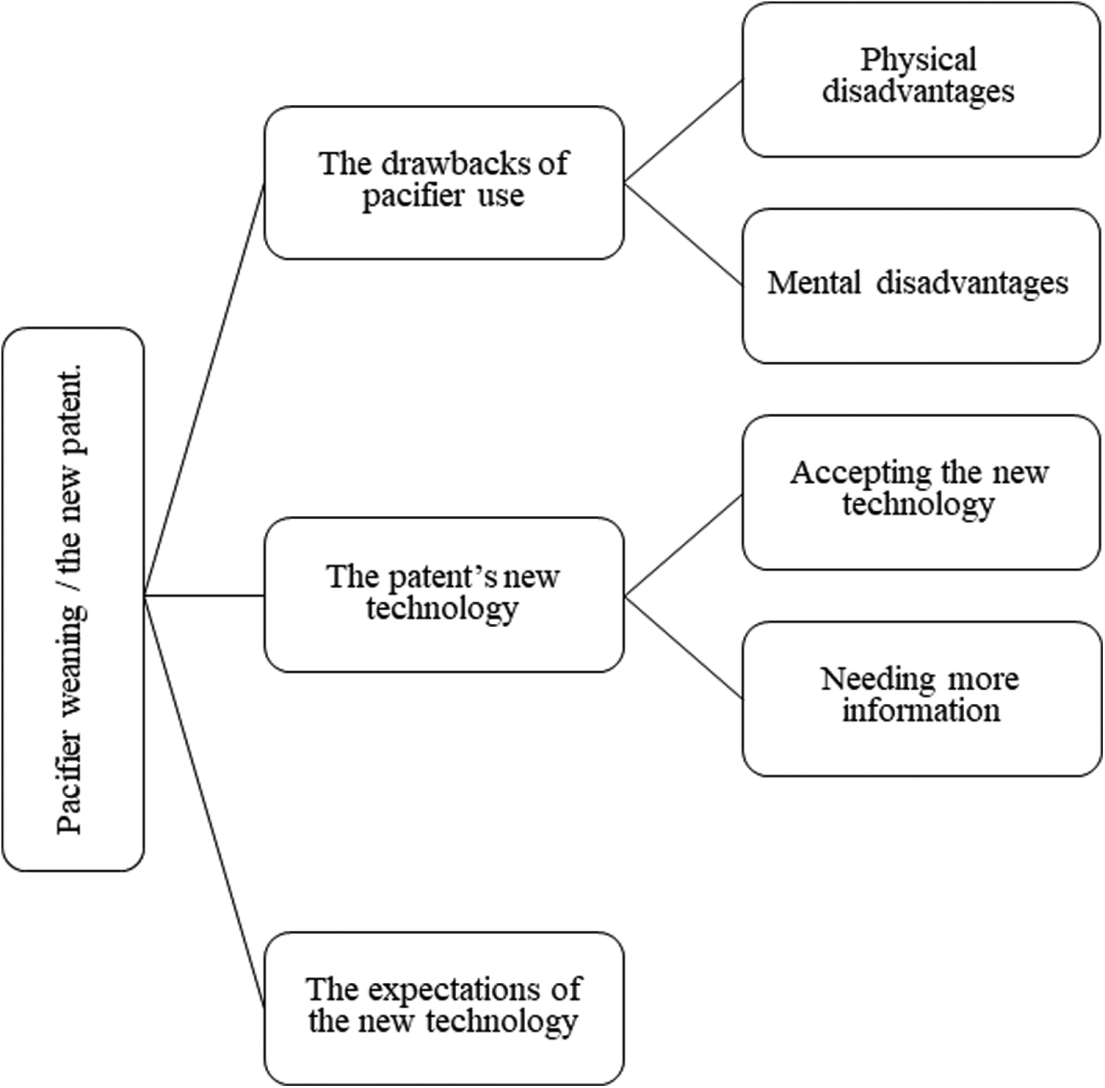
Thematic map of themes and subthemes.

Data from the interviews were categorized into three themes: (1) the drawbacks of pacifier use, (2) the patent's new technology, and (3) the expectations of this new patent. Two subthemes were identified for the first theme: physical and mental disadvantages. Two subthemes were also recognized for the second theme: accepting the new technology and needing more information. No subthemes were identified for the last theme. Figure 3 illustrates the themes and subthemes.

The following sections discuss the themes and include relevant interview excerpts that support the findings. As expected with qualitative data, some extracts connect to more than one theme. However, the extracts highlighted in this paper represent the most applicable theme or subtheme.

#### 3.2.1. The drawbacks of pacifier use

The participants discussed many disadvantages of pacifier use, which were categorized into two subthemes. The first subtheme is the physical disadvantages of pacifier use. The participants discussed these drawbacks based on their specializations and provided the following feedback:

Pacifiers cause a lot of sicknesses, such as infections. It causes bacterial and fungal infections. A baby plays everywhere inside the house and outside the home. When he throws his pacifier on the floor, on the table, or any surface, the rubber part of the pacifier will hold bacteria and germs. This will cause patches and spread thrush in the baby’s mouth (Pediatrician 1).

Young children may suffer from middle ear inflammation if they use a pacifier. The continuous sucking on a pacifier makes throat secretions move to the middle ear (Family Doctor 2).

I believe that pacifier use causes teeth problems. It affects the normal development of the teeth. It may cause crooked teeth and then malocclusion. It might distort the jaws (Dentist 3).

I don’t like a pacifier. It affects [breastfeeding]. My daughter does not want my milk that much like before [she used a pacifier]. She used to be breastfed many times during the day, but now she breastfeeds only two or three times (Mother 1).

In addition to the physical drawbacks, the mental disadvantages of pacifier use are a second subtheme. The mothers and psychologists were the participants who discussed this subtheme more than the others, as indicated by the following interview excerpts:

[Pacifier use] causes loss of appetite. If my baby uses the pacifier, he does not care about food. Whatever I do and whatever I cook for his meal. I mean, in a delicious way, he does not care. He eats a little, and that’s it. It is so frustrating … There is also the problem of sleeping. Every time he must suck his pacifier to be calm and sleep, the problem is when his jaw is relaxed, the pacifier falls off, and then he wakes up, and I must put the pacifier back into his mouth. Otherwise, he will cry. I do this endless task at night. It is very exhausting (Mother 2).

I know that a pacifier is a solution to make a child quiet. But the problem is that a child wants it all the time, and he depends on it, and if he does not get it, he will be very hyperactive. Imagine if I forget to take [the pacifier] with me when we are going out. He will be mad! (Mother 3).

Although pacifier use has some advantages, I think the negatives [outweigh] the [positives]. One of my patients is three years old, and I don’t see her without a pacifier in her mouth. The problem here is that she doesn’t speak. She only points with her finger. [The] pacifier, in her case, causes a decline in pronunciation and communication skills (Psychologist 2).

Some participants indicated a few pros of pacifier use, but all participants stressed the cons. The data reflect a rejection of pacifier use because it probably leads to several problems related to the child's health, including physical or mental issues.

#### 3.2.2. The patent's new technology

The current patent's new technology (Prevents Getting Used to Pacifier Baby) was introduced to each participant by presenting Figures 1, 2. A physical model of the pacifier was also shown to the participants. Each part of the patent was explained extensively. Some respondents had clarification questions, and adequate answers were provided. Even though most of the participants accepted the new technology, a few asked for additional clarification. The first subtheme is accepting new technology, as reflected in the following excerpts:

When a baby uses this new pacifier, he will not suck it because it shocks him. If this works well, the child will leave the pacifier forever (Pediatrician 2).

I think this technology will be sufficient because it links pleasure with pain. I mean, we know that a pacifier is fun for a child, but this shocking pacifier will change this belief in the baby’s mind and [make] him hate it because it is painful (Psychologist 1).

I believe this patent will be helpful for the child and his mother. Pacifier use is a bad habit, but this new patent will cause a tiny shock to the baby, and this will make him refuse any pacifier (Family Doctor 3).

When I use electric dentist tools, like the dental drill, many children do not like its sound and jiggling, and some refuse to use it in their mouth. It is shocking inside their mouths, so I think this patent technology will create the same negative feelings (Dentist 1).

This patent is better than cutting the pacifier rubber side. With my first child, I had to follow a method that some mothers told me about cutting the rubber side of the pacifier bit by bit from time to time. Eventually, my child doesn’t like it and leaves it, but I have two problems with this method. First, it takes a very long time. Second, I open the part my son sucks at, which may create a suitable environment for germs, but this new technology looks promising (Mother 2).

Despite the positive perspectives of the patent, a few participants were not decisive about it. Hence, the second subtheme is that more information is needed about the patent, as indicated by the following interview excerpts.

I am not sure about it. I think this technology needs to be used only with kids [who] use pacifiers extensively and are emotionally attached to them (Mother 1).

I think it is helpful, but this technology needs to be investigated, and we need to try it first and see if there is any harm from the electric shock on the child (Pediatrician 2).

Any new technology has its positives and negatives. Most kids stop using pacifiers by themselves at ages two to four, so I don’t think this new pacifier will be needed for all the kids. Only the ones who need help to break this habit need this new technology. I guess the vibrations that this pacifier makes would help in pacifier weaning (Family Doctor 1).

Thus, most participants formed a positive view of the new patent when it was introduced to the sample and were open to it. As expected, some participants had doubts about the patent and thought it should be given to children who use pacifiers extensively and continuously.

#### 3.2.3. The expectations of the new technology

The last theme identified by the data was the participants’ expectations about the patent, as reflected in the following interview excerpts:

Some mothers cannot stop the pacifier use habit quickly. I hope this electric pacifier helps them more than their funny methods. I believe it will help them better (Mother 2).

I think using it in the first few months would be beneficial, I mean, before the child gets older and gets used to it, but when this pacifier is manufactured for commercial purposes, it should be designed in all sizes, even for two- or three-year-old kids (Psychologist 3).

This new technology will be a new device that can be added to all other devices and tools related to babies and toddlers, but it is essential to use this new pacifier occasionally, sterilize it and keep it clean, and change it from time to time. Also, I think it is important to manufacture it using materials that do not harm a child’s health (Dentist 2).

The child and his parents need to know the disadvantages of pacifier use. I think this patent will be helpful, but the parents should know everything about it (Pediatrician 3).

The participants’ expectations focused on the benefits of the new technology. They hope the new patent works well and its advantages and disadvantages will be delineated.

## 4. Discussion

This qualitative study introduced a patent that involved new technology to prevent pacifier use and to help with pacifier weaning. Thematic analysis was adopted, and the data were categorized into themes and subthemes, including the health problems associated with pacifier use, the new patent's technology, and the expectations of this new patent. Although pacifier use has some benefits, it is an issue for mothers and professionals treating babies or toddlers who use pacifiers.

Like the current study, some studies have investigated and evaluated the advantages and disadvantages of pacifier use. For instance, in their qualitative data analysis, Rocha et al. (44) found that although pacifier use is good for the maternal/infant experience, it causes issues with breastfeeding. The researchers also discussed other long-term concerns, including reliance on pacifier use and its effect on children's teeth. Likewise, Eidelman (45) concluded that pacifier use helps reduce the risk of SIDS and generally soothes and calms the baby. However, continued pacifier use should be discouraged because it may lead to infections, middle ear inflammation, and distortion of the jaws [see also Warren et al. (13), Rovers et al. (12), Caruso et al. (46)].

Inventions have aimed to help pacifier weaning, such as Bashir’s (28) patent (Number WO2016174381A1) and Parker and Ramundo’s (30) patent (Number US8298263B2). However, the present patent (Figures 1, 2) is different from the other patents in three ways.

First, the current patent uses only a two-volt lithium battery (or a four-volt lithium battery) to create an electric shock when the child's saliva touches the exposed wires on the pacifier nipple. From the ethical perspective, a comparison between electroconvulsive therapy (ECT) and the current patent will be discussed. ECT is a safe and reliably effective treatment for a number of mental health conditions and behavioral disorders (47). In contrast, the current patent is not a treatment—it is a device that may deter a child from using a pacifier. However, both (ECT and the current patent) depend on the electric shock. Abrams (48) stated, “ECT as a procedure is ethical”, and he also stated, “that the response to ECT in children …  is no different from that obtained in adults: excellent”. There are studies reported the use of ECT on children for many reasons like mental issues, bad behaviours, disorders, and mood disorders (49–53) which confirms that an electric shock is ethically acceptable. Considering that ECT uses voltage ranging from 70 to 120 volts (32), while the current patent uses only 2–4 voltage. Therefore, if the ECT got ethical approval, uses high voltage, and was applied to people of all ages, including children, then this is even more the case with the current patent, i.e., it is ethical. Furthermore, the current patent meets the four main ethical principles of medical care: beneficence (doing good), non-maleficence (not doing harm), autonomy (right to refuse or accept treatment), and justice (equality of opportunity) (54).

In addition, electric shock is used as a deterrent for bad habits in adults. Kreitmair (55) discussed the benefits of using a Pavlok. It is a wrist-worn wearable device that vibrates and uses electrical stimulus—up to 450 volts—to prevent and stop bad habits such as smoking cigarettes, being addicted to smartphone use, nail-biting, or eating candy [see also Pavlok 2 (56)]. Using Pavlok is also ethical (57). Furthermore, the electric shock has other daily benefits, including its use in electric toothbrushes and electronic message devices. This shows that the current patent may be helpful in pacifier use.

Second, even though the current patent has already been registered with the Saudi Authority for Intellectual Property (Patent Number SA10609), mothers and health practitioners reviewed it to add more credibility to the patent. Third, this patent is a single piece that takes the shape of a regular pacifier. It is simple and easy to use, its battery can be changed, and its electric shocker is replaceable if it does not work. This pacifier can also be made commercially available in various sizes and colors [this latter is consistent with the fourth ethical principle of medical care, which is justice—equality of opportunity—see Beauchamp and Childress (54)].

In short, this patent is another attempt that might help break the habit of pacifier use. If children stop using pacifiers, their physical and mental health may be improved, and their parents will have peace of mind and be relieved from all the negatives of pacifier use.

### 4.1. Implications for practice

The current findings have numerous implications for parents and health practitioners, especially those dealing with babies and young children who use pacifiers. The results suggest that there are drawbacks to pacifier use. In other words, it may affect children's health, including mouth infections, middle ear inflammation, dental problems, distorted jaws, avoidance of or diminished preference for breastfeeding, loss of appetite, interrupted sleep, and a decline in pronunciation and communication skills. Even though research has demonstrated that pacifier use provides some benefits (1, 3), literature corroborates the current findings and explains that avoiding pacifier use is better [such as World Health Organization (7), Vogel et al. (9), Rovers et al. (12), Jenik and Vain (6), Kronborg and Vaeth (10), Almqvist-Tangen et al. (11)]. This is supported by the fact that several patents and methods have been invented to help pacifier weaning (30, 28), including the current patent.

Therefore, the recommendation is to introduce this new technology to a broader range of parents and health practitioners and encourage them to try this current patent. This patent may help children stop pacifier use in the short term and may protect their physical and mental health for a long time.

### 4.2. Strengths, weaknesses, and future directions

Some study limitations are essential when interpreting findings and establishing a direction for further research.

First, the current data are qualitative, and thematic analysis was adopted to explore the intricacies of the meaning of the data. Thus, this exploratory investigation is a pilot study or an initial step that paves the way for additional studies. Especially with the case of a patent—as is the case with the current device—it is curial to present a profound description of the patent (as was conducted with the current sample of mothers and health practitioners) before experimenting this patent [see Dias and Almeida (58)]. Causal relationships between, for example, the current patent and pacifier weaning could not be examined. However, this relationship can be investigated by an experimental study that targets these two factors in the future.

Second, although the current sample is diverse regarding the participants’ professions, the sample does not include all possible health practitioners who treat babies and children. In particular, an Ear, Nose, and Throat—ENT specialist (i.e., an otorhinolaryngologist) would be the best expert to provide insights on problems related to pacifier use, especially since pacifier use may lead to recurrent acute otitis media (12). Hence, adding otorhinolaryngologists to the sample in future research would help investigate children's health related to the ear, nose, and throat. Furthermore, as for the use of electric shocks, neurologists and mental health specialists can offer valuable insights into the benefits and potential risks of such a patent.

Third, the current study focused only on one patent. In contrast, other patents have been invented to stop pacifier use, such as Bashir’s (28) patent and Parker and Ramundo’s (30) patent. Conducting a comparative study will help identify the best available technology or the most relevant patent for pacifier weaning. This type of study will also show the advantages and disadvantages of these patents and their similarities and differences. This would open the door to invent more advanced technologies or patents to achieve the goal of pacifier weaning.

To the best of our knowledge, despite these limitations, this is the first study to discuss the usefulness of the patent (Number SA10609). Instead of making this new technology unavailable, this paper provides an in-depth evaluation of all the details of this pacifier-weaning technology. It presents the opinions of mothers and child health experts about the technology.

## 5. Conclusions

The current qualitative data and the thematic analysis reflect three key themes: (1) the drawbacks of pacifier use, (2) the new patent's technology, and (3) the expectations of this new patent. The findings reveal that although pacifier use has some benefits, the disadvantages outweigh the advantages. However, further investigation is needed to test the impact of the current patent on pacifier weaning.

## Data Availability

The raw data supporting the conclusions of this article will be made available by the authors, without undue reservation.
